# Accuracy of the Masimo Pronto-7^®^ system in patients with left ventricular assist device

**DOI:** 10.1186/1749-8090-8-159

**Published:** 2013-06-24

**Authors:** Christian S Bruells, Ares K Menon, Rolf Rossaint, Andreas Goetzenich, Michael Czaplik, Norbert Zoremba, Rüdiger Autschbach, Gereon Schaelte

**Affiliations:** 1 Department of Anesthesiology, University Hospital Aachen, Rhenish Westphalian Technical University (RWTH) Aachen, Pauwelsstr.30, 52074, Aachen, Germany; 2 Clinic for Cardiac, Thoracic and Vascular Surgery, University Hospital Aachen, Rhenish-Westphalian Technical University (RWTH) Aachen, Pauwelsstr. 30, 52074, Aachen, Germany; 3 Department of Intensive Care and Intermediate Care, University Hospital Aachen, RWTH Aachen University, Pauwelsstr, 30, Aachen 52074, Germany

**Keywords:** Perioperative care, Circulatory assist devices, Blood transfusion, Emergency, Patient safety

## Abstract

**Background:**

The Masimo Pronto-7^®^ calculates hemoglobin (Hb) values using the pulsoximetry technique and a variety of mathematical algorithms analyzing the pulse waveform. Although this system has demonstrated a high level of accuracy in average patients, the performance might be altered in special patient populations. Regarding patients with left ventricular cardiac failure, a rotary blood pump generates a constant, continuous, non-pulsatile flow to improve effective cardiac output. Due to this alteration in both, blood flow and arterial blood pressure we hypothesized a reduced accuracy of the Masimo Pronto-7^®^ to detect Hb in patients with left ventricular cardiac failure. To test our hypothesis, we evaluated the Pronto-7^®^SpHb system in outpatients after continuous-flow-left ventricular assist device (cf-LVAD) implantation (HeartMate II, Thoratec).

**Methods:**

21 cf-LVAD outpatients from the Clinic for Cardiac, Thoracic and Vascular Surgery were investigated during routine follow up examinations. After venous blood samples were drawn, the Pronto-7^®^ sensor was attached to one randomly selected finger of one hand. The collected SpHb data were compared with Hb values measured by our central laboratory. The difference between the methods was determined using Bland – Altman analysis. The study was registered in the DRKS (DRKS00004415).

**Results:**

In all cf-LVAD patients evaluated, the Pronto-7^®^ successfully detected SpHb values. Using Bland – Altman analysis, a bias of 0.14 g/dl (95% upper and lower limits of agreement ± 2.76 g/dl) was calculated.

**Conclusion:**

The Pronto-7^®^ overestimated the actual Hb value in cf-LVAD outpatients with the HeartMate II. Due to this, we conclude that the system is suitable for screening in routine examinations and further analysis can be performed if needed. However, its use as an emergency tool is questionable because of the increased inaccuracy when Hb values are critically low.

## Background

Performance of pulse oximetry has improved during the last few years. Indeed, current pulse oximetry devices have reached a high level of validity and reliability, even in patients with altered or hampered pulse wave curves [1]. The development is owed to an increasing number of wavelengths used for light emitting diodes and improved mathematical algorithms that allow higher stability of the signal and validity of the interpretation. Several new analyzing tools, including pulse oximetry based hemoglobin (SpHb) monitoring, have recently been established and tested in different clinical settings, such as acute hemorrhage [2], obstetrics [3], conditions of hemodilution [4] and on mixed ICU patient populations [5].

Concomitant to this huge step in pulse oximetry performance, challenges for acute measurements for these systems have remained, especially in cardiac surgery patients. Several devices have been introduced to support cardiac function or to take over the complete left, right or both cardiac outputs. Implanted devices in patients suffering from severe cardiac failure are generally classified into pulsatile or continuous flow (cf) generating systems with advantages and limitations for both systems. Higher long-term survival rates at two years are currently being reported for patients supported by continuous flow systems [6]. Importantly, adverse events and the need for device replacement are less frequent in patients with continuous flow systems, which may favor their use over pulsatile systems. However, the risk of bleeding exists in both systems, with the rate of occurrence for packed red blood cell transfusions up to 81% [6].

As a function of design, pulsatile LVAD are more similar to the normal, physiologic cardiovascular function, which is mainly characterized by the beating heart with accelerating and decelerating flow rates. Conversely, cf-LVAD generate a continuous flow of blood with no detectable changes in flow rate. These devices are comparable to the cardiopulmonary bypass with no blood pressure curve, rather they are characterized by the absence of systolic and diastolic pressures. Nevertheless, in many patients some low-grade basal left ventricular function remains or is regained after device implantation, generating an ‘on top’ pulse wave.

The absence of a pulsatile flow in patients with cf systems is a challenge for pulse oximetry based Hb detecting systems and the implemented mathematical algorithms. To test the accuracy of the Pronto 7^®^ system, we measured SpHb values in 21 cf-LVAD patients with implanted HeartMate II devices and compared these values to the Hb measurements taken from venous blood samples. Due to the convenience, speed and improved performance of pulse-oximetry, it is important to know whether this device can accurately detect Hb in patients with cf-LVAD devices. Moreover, given the high number of bleeding incidents in cf-LVAD patients, accurate SpHb measurements are crucial in emergency settings. We hypothesized that the accuracy of the Masimo Pronto-7^®^ system for SpHb measurements may be reduced in patients with cf-LVAD devices due to the absence of pulsatile blood flow.

## Methods

21 Caucasian patients (13 male, 8 female) from the specialized outpatient division for assist device patients of the Clinic for Cardiothoracic and Vascular Surgery of Aachen University Hospital were investigated. The ethics committee of the medical faculty of the RWTH Aachen waived the requirement to obtain informed consent from patients due to the non-invasive nature of the study (EK 223/12). Venous blood samples were taken from the median cubital vein as part of routine follow up examinations based on the established protocol for cf-LVAD patients. However, in one patient who was being treated in the intermediate care unit due to recent bleeding, the blood sample was obtained from the central venous line. After the vw ^®^ SET**^®^** Pronto-7^TM^ Puls CO-Oximeter generating several wavelengths in a range of 500 nm-1300 nm to detect SpHb values besides functional oxygen saturation values (SpO_2_), pulse rate (PR), and Perfusion index (PI). The installed software calculates SpHb values based on the pulse-waveform, the detailed mathematical formulas are not publically available. The Pronto-7^®^ was set to the mode that allowed a higher acceptance of unusual pulse wave forms but linked with a 10% higher standard deviation of differences, (i.e. 68% of the SpHb values have a lower difference than 1.1 g/dl to a Hb value, instead of 1 g/dl in the standard operating mode). The finger clip (medium size) was placed randomly at one of the three fingers (Dig II, III, or IV) of one hand. Five attempts were made to obtain the SpHb measurements. If the system could not calculate a SpHb, the finger clip was replaced anew to exclude misplacement. Finger nails were free of nail polish to reduce any potential measurement errors. The first SpHb value displayed was noted as well as PR, SpO_2_ and PI. All blood samples (EDTA) were investigated in the hospital’s central laboratory facility following standard clinical procedure. Blood pressure (RR) was measured manually after the venous blood draw and pulse oximetry measurements.

### Statistical procedures

Mean and standard deviations of SpHb, Hb, PR, PI were calculated. The difference between SpHb, and Hb values was analyzed based on the methods published by Bland and Altman [7]. The limits of agreement were estimated by calculating the mean difference (bias) and the standard deviation (SD).

The upper and lower limits were defined as bias ± the range in which 95% of the differences between the methods were expected to occur. The confidence intervals for the limits of agreement were defined by the term $\sqrt{{3SD}^{2}/n}$ where n is the sample size. The CI for the biases were calculated based on the sample size by the term $\sqrt{{SD}^{2}/n}$. Linear regression analysis was used to detect dependencies between PI, PR, RR and the relative differences between SpHb and Hb values. The methods described above have been used before by our group [8]. All statistical calculations were done using GraphPad Prism version 5.00 for MacOS, GraphPad Software, San Diego California USA,) and SPSS 20 (IBM Corporation, Armonk, New York, USA).

## Results

### Patients

The Pronto-7^®^ system was able to acquire data in 18 patients on the first attempt. In one patient, three attempts were needed, in another one five attempts were necessary to obtain a SpHb value. One patient investigated was treated for sub-acute bleeding and was recovering in the intermediate care unit, having received two units of red packed blood cells two hours before. Patient characteristics are given in Table 1. The patient characteristics concerning age, BNP levels, PR, systolic RR and PI did not differ significantly. In four patients no diastolic blood pressure could be measured. No adverse events occurred in any of the patients during the study.

**Table 1:** Patient characteristics of the left ventricular assist device patients investigated

	**Absolute**	**Male**	**Female**
Age (mean±SD)	62.9±10.1	65.77±8.02	57.1±11.90
Systolic blood pressure (mmHg, mean±SD)	103.3±11.65	104.5±13.16	101.4±9.4
BNP (mean±SD)	1025.05±874.74	1217.5±979.53	695.14±259.52
Pulse rate (per minute, mean±SD)	68.95±13.12	67.46±14.49	71.71±9.5
Pulse index (mean±SD)	2.63±2.98	3.2±3.42	1.56±1.05
Comorbidities –n (%)			
Coronary artery disease	18 (90%)	12 (92%)	6 (86%)
Arterial hypertension	13 (65%)	8 (62%)	5 (71%)
Diabetes	6 (30%)	5 (38%)	1 (14%)
Hyperlipidemia	8 (40%)	6 (46%)	2 (29%)
Renal failure	10 (50%)	8 (62%)	2 (29%)
Stroke	3 (15%)	1 (8%)	2 (29%)
Peripheral vascular disease	7 (35%)	5 (38%)	2 (29%)
Pacer /ICD	7 (35%)	5 (38%)	2 (29%)
Atrial fibrillation	1 (5%)	1 (8%)	0
Valve dysfunction			
Aortic valve stenosis	4 (20%)	3 (15%)	1 (14%)
Aortic valve insufficiency	1 (5%)*	1 (8%)	0
Mitral valve insufficiency	2 (10%)	1 (8%)	1 (14%)^#^
Tricuspidal valve insufficiency	0	0	1 (14%)^#^
Pulmonary hypertension	8 (40%)	5 (38%)	3 (43%)
Acute hemorrhage	1 (5%)	1 (8%)	0
Chronic hemorrhage	1 (5%)	1 (8%)	0
Chronic obstructive pulmonary disease	5 (25%)	4 (31%)	1 (14%)

*combined vitium (stenosis and insufficiency) in one patient, # combined mitral tricuspid insufficiency in one patient. BNP= Brain natriuretic peptide. Percentages are given between male/female patient population or in between the sex groups.

### Differences in Hb and SpHb values

**Figure 1:**
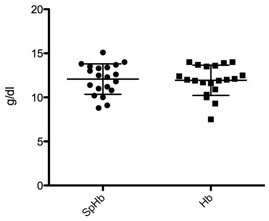
Distribution of absolute values of SpHb and Hb values from the investigated left ventricular assist device patients including mean and standard deviation.

**Figure 2:**
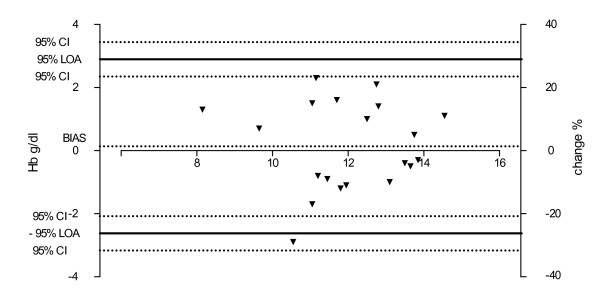
**Bland-Altman plot for differences between SpHb and Hb values.** The upper and lower Limits of agreements (LOA) were calculated as the bias ± 2 standard deviations.

The mean Hb value of the patients was 11.94 g/dl (SD ±1.71), with a range between 7.5 g/dl and 14 g/dl. The mean SpHb values as measured by the Masimo Pronto-7^®^ was 12.08 g/dl (SD ±1.73 g/dl), with a range between 8.8 g/dl and 15.1 g/dl (see Figure 1). The mean difference (i.e., bias) between the SpHb values measured with Pronto-7^®^ and the Hb measured in the venous blood sample was 0.14 g/dl (95% CI: ± 0.31). The upper and lower limits of agreement were calculated as -2.62 g/dl and 2.89 g/dl (95% CI: ± 0.55) (Figure 2). Regarding the relative differences between SpHb and Hb values, the bias was 1.20% (95% CI: ± 2.8) with upper and lower limits of agreement ranging between -23.34% and 25.74% (95% CI: ± 4.85), respectively (Figure 2). Linear regression models calculated from PI, PR and RR values and the relative differences between SpHb/Hb values revealed no dependency of these parameters to the relative differences (PI: R^2^ = 0.001, PR: R^2^ = 0.032, RR: R^2^ = 0.01).

## Discussion

Using Bland – Altman analysis, our study revealed a bias of 0.14 g/dl (or 1.2%) between the SpHb and Hb values with limits of agreement between 2.62 g/dl and 2.89 g /dl (23.3% and 25.74%). We could not detect a correlation between clinical parameters as PR or RR and SpHb accuracy. A detailed discussion of the findings follows.

For SpHb measurements, a variety of studies have been performed to gain insight about the accuracy of this novel technique in different clinical settings [2-4,9-11]. The accuracy of SpHb measurements in stable patients without acute hemorrhage or cardiovascular insufficiency is remarkable [1,5], defining the Pronto-7^®^ as a tool of standard care procedure. We investigated the use of this device on a patient subset with a physiological perturbation that could likely impact the measurement of Hb. Given that our subjects lacked pulsatile blood flow, we expected this to likely influence both flow and pulse waveform characteristics. Nevertheless, in all our patients, the device successfully detected an Hb value, indicating that the remaining contractions of the left ventricle generated a sufficient enough pulse wave that could be analyzed by the implemented algorithm of the Pronto-7^®^.

### Patients

The patients we investigated were all in stable clinical conditions as indicated by ECG, pulse rate, systolic blood pressure and hemoglobin values (see Table 1), except for one patient that was treated in the intermediate care unit after an acute hemorrhage. The incidence of ischemic cardiac disease was elevated in our patient population compared to the patient population described by Slaughter et al. who reported ischemic heart failure of 66% [6]. Mean Hb values of the patients (11.94 g/dl (SD ±1.71)) are slightly lower than the normal range for our laboratory (12-16 g/dl). The one patient suffering from acute hemorrhage that required surgical intervention and transfusion had the lowest Hb values (7.5 g/dl), and remained stable afterwards. We were able to successfully obtain SpHb values from all 21 patients which is a lower rate of failure (0.0%) than previous studies who could not detect a SpHb in <2.5% of a mixed patient population [12] or 8% of emergency ward patients [13]. Nevertheless, in two of our patients, several attempts had to be made to successfully obtain values.

### Accuracy of the SpHb measurements

Our results with a bias of 0.14 (SD ± 1.41) g/dl regarding absolute difference are comparable to different clinical studies that show the same low levels of bias [4,5]. Frasca et al. investigated intensive care patients, found a bias of 0.0 g/dl [5], while Berkow et al. detected a bias of -0.1 g/dl during spine surgery [2]. Hemodilution in healthy volunteers did not affect the accuracy with a bias of -0.15 g/dl [4]. In contrast, much higher biases were found in more heterogeneously distributed patients (0.56 g/dl) [12] or in patients with acute hemorrhage (up to 1.0 g/dl) [14] respectively. In the mode of maximal sensitivity of healthy volunteers, the manual describes an SD of ±1.1 g/ dl when values were between 6-18 g/dl. Our study detected a SD of the bias of ± 1.41, which is higher. These findings could be interpreted as high or negligible, depending on the clinical situation. Therefore we calculated the relative bias (1.2%), underlining the small variety of SpHb-Hb differences. Even more interesting for the assessment of accuracy besides the bias are the upper and lower limits agreement, (i.e. the range in which 95% of the differences between the methods are expected to occur). In this trial those limits ranged between -2.62 g/dl and 2.89 g/dl. This range exceeded the limits given for FDA approval in the user manual (2 g/dl) and are lower than the findings of Miller in patients during spine surgery [10], but higher than Berkow et al. reported in their patient population [2]. Interestingly, in cardiac surgery patients, Nguyen revealed a wide range of limits of agreement (between -4.6 g/dl and 2.1 g/dl) [11]. These values were influenced by the software release, as a consequence we used the latest software update available. The updated software possibly explains the increased accuracy of our findings. Nguyen et al. concluded that based on their findings, the SpHb system cannot (yet) be recommended [11]. This conclusion is shared by Gayat and colleagues based on the mixed patient population study with nearly 300 patients [12]. In addition to the absolute values, we calculated the relative values to allow a better interpretation and weighting of these differences. The calculated limits of agreement (LOA; -23.34% and 25.74%) support the conclusions drawn by the study of Ngyuen and Gayat: that the Pronto-7^®^ is not recommended for patients with cf-LVAD in its current state [11,12].

**Figure 3:**
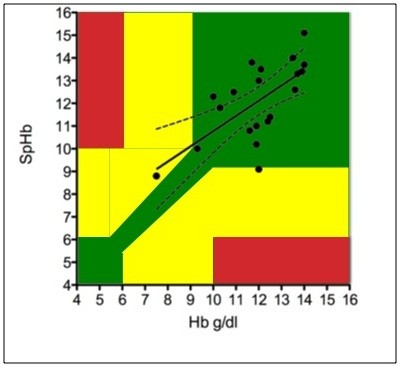
**Error grid analysis, redrawn after **[15]** using a linear regression plot between SpHb and Hb values (g/dl).** The dotted lines indicate the 95% prediction bands, in which 95% of the data points are expected to fall. The graph is consisting of three zones (red, yellow, green), in which the bias of the measurements results in different clinical decisions. While in the green zone the bias is without further therapeutic consequence, the errors lying in the yellow or red zone may lead to major therapeutic errors.

The lack of accuracy is problematic for anemic patients since these are the patients that stand to benefit the most from a quick and reliable diagnosis. The following example from our own study illustrates the importance of accurate readings in anemic patients. Our patient that suffered from an acute hemorrhage had a Hb value of 7.5 g/dl as measured from the venous blood sample; as such, a transfusion of red packed blood cells is indicated. The SpHb value obtained from the Massimo Pronto-7^®^ differed by 17% (8.5 g/dl SpHb). Importantly, these values are close enough to a “safe” value, where no transfusion might be considered. Furthermore, the absolute difference between the Pronto-7^®^ and the measured venous sample (1 g/dl) is still within the range given by the manufacturer. This situation clearly emphasizes the dilemma of the utility of the device at low Hb values. Morey and colleagues [15] created a graph consisting of three zones (red, yellow, green) (see Figure 3), in which the bias of the measurements can result in different clinical decisions, his figure is based on a publication regarding SpHb device accuracy [4]. When values are within the green zone, the bias is without further therapeutic consequence, the errors lying in the yellow or red zone may lead to major therapeutic errors. The authors defined these zones according to the 2006 practice guidelines for transfusion developed by the American Society of Anesthesiologists Task Force on Perioperative Blood Transfusion and Adjuvant therapies and a possible deviation of 10% (*i.e.,* 1 g/dl at Hb 10 g/dl). Using this graph (Figure 3), we conclude that the accuracy of this device is unacceptable for diagnostic use to base therapeutic interventions in patients with cf-LVAD implants. However, given these findings, the Pronto-7^®^ could be an acceptable and convenient preliminary screening device to identify cf-LVAD patients that may be at risk for anemia and in need of further testing.

### Comments on the study methods and design

There are several points to address regarding our research design and methods. From a basic science perspective, these issues may be viewed as limitations. However we see them as strengths because they replicate clinical conditions of use. First, we accepted the first SpHb value the device displayed in our study and did not repeat the measurements to examine the data variance. We sought to use a clinical approach, where a reasonable measurement and not the expected one is used. Another strength is that we used the mode with a wider range of acceptance for pulse wave abnormalities (so called ‘maximal sensitivity’) thereby tolerating a larger bias and SD. Using this mode would be the appropriate setting for patients with cf-LVAD given that they lack the needed pulsatile flow for the device to function. We saw that enhancing the sensitivity would enable the device to detect the almost negligible pulse waves generated by these patients. Obtained LOA were even higher than they should be for the FDA approval (2 g/dl). Lastly, due to our clinical aim, a typical clinical scenario was selected for this practical test.

While we see many of the above mentioned aspects of this study as strengths, we realize there are limitations. First, our study population of only 21 patients may have influenced our limits of agreement, which might be smaller by investigating larger sample sizes. Nevertheless, our data are in line with prior publications [11]. Second, blood samples were collected from the median cubital vein and not from an arterial catheter from the same hand as described in other publications [2,11]. However, we do not expect this to have a profound influence on our findings.

## Conclusion

The Pronto-7^®^ system could detect a SpHb value in all patients with a cf-LVAD, despite their characteristic lack of pulsatile flow. Accuracy of the device in the tested patient population was hampered by a wide range of limits of agreement which make the Pronto-7^®^ inappropriate in patients with cf-LVAD presenting with anemia, however, this device might be sufficient as a screening method used in routine exams for patients with cf-LVAD with additional need for confirmation on a case to case basis.

## Abbreviations

cf-LVAD: Continuous flow left- ventricular assist device; Hb: Hemoglobin; SpHb: Puls oximetry based hemoglobin; SpO2: Oxygen saturation; PR: Pulse rate; PI: Perfusion index.

## Competing interests

The authors declare that they have no competing interests.

## Authors’ contributions

CB and AM: study design, measurements, manuscript drafting, RR and RA: study design, critical revision, AG: measurements, MC and NZ: data analysis, statistical work. All authors read and approved the final manuscript.
